# Chromosome-Level Genome Assembly of *Discogobio brachyphysallidos* (Teleostei, Cyprinidae) and Population Genomics of the *D. brachyphysallidos* Complex: Impacts of Geological and Climate Changes on Species Evolution in Southwest China

**DOI:** 10.3390/ijms252413462

**Published:** 2024-12-16

**Authors:** Lan-Ping Zheng, Li-Li Wu, Hua-Ying Sun

**Affiliations:** College of Chinese Materia Medica, Yunnan University of Chinese Medicine, Kunming 650500, China; wulili@ynucm.edu.cn (L.-L.W.); sunhuaying@ynucm.edu.cn (H.-Y.S.)

**Keywords:** nanopore, Hi-C, resequencing, Cyprinidae, karst region, Southwest China

## Abstract

The genus *Discogobio* is distributed in the eastern three rivers on the Yunnan–Guizhou Plateau and its adjacent regions, located to the southeast of the Qinghai–Tibet Plateau. Its origin and evolution are likely influenced by the uplift of the Qinghai-Tibet Plateau. However, the historical impact of geological events on the divergence and distribution of this fish group has not been fully elucidated. In this study, we successfully assembled a chromosome-level genome for *Discogobio brachyphysallidos*, which is approximately 1.21 Gb in length with a contig N50 of 8.63 Mb. The completeness of the genome assembly was assessed with a BUSCO score of 94.78%. A total of 30,597 protein-coding genes were predicted, with 93.92% functionally annotated. Phylogenetic analysis indicated that *D. brachyphysallidos* was closely related to *Labeo rohita*, and the divergence of the subfamily Labeoninae coincided with the significant uplift events of the Qinghai–Tibet Plateau. Additionally, we analyzed 75 samples of *D. brachyphysallidos* and *D. yunnanensis* from five populations, yielding 1.82 Tb of clean data and identifying 891,303,336 high-quality SNP sites. Population structure analyses indicated that the populations were clustered into five distinct groups, demonstrating significant genetic differentiation among them and the presence of cryptic species within this genus. Analyses of linkage disequilibrium decay and selective sweep indicated that the Pearl River population exhibited relatively higher genetic diversity compared with the populations from other drainages, and none of the populations showed evidence of expansion. Notably, the two population declines coincided with the early Pleistocene and Quaternary glaciation. It can be assumed that the geological movements of the Qinghai–Tibet Plateau and the Quaternary glaciation contributed to the decline in *Discogobio* populations and shaped their current size. The population genomics results showed that the present distribution pattern of *Discogobio* was the outcome of a series of geological events following the uplift of the Qinghai–Tibet Plateau. This study reconstructed the geological evolutionary history of the region from the perspective of species evolution. Furthermore, our study presents the first genome-wide analysis of the genetic divergence of *Discogobio*.

## 1. Introduction

The subfamily Labeoninae (Teleostei, Cyprinidae) is a benthic species adapted to the rapid waters in Asia and Africa [1]. This group of fishes possesses an inferior mouth and has developed diverse oral morphology, which make its taxonomy confusing. Past studies of subfamily Labeoninae have been focused on the taxonomy and phylogeny [1,2,3]. In recent years, the results of molecular phylogeny have gradually demonstrated the phylogenetic relationships of the subfamily and its subdivisions [3]. Subfamily Labeoninae was mainly distributed in Asia and Africa, and Asia is the main center of origin and evolution of this group of fishes [4]. Specifically, Labeoninae was distributed to the south of the Qinghai–Tibet Plateau in East Asia, and hence it is likely that the evolution of these species in this area was influenced by the uplift of the Qinghai–Tibet Plateau [5].

The genus *Discogobio* is a member of subfamily Labeoninae, and it is characterized by a sucker disc on the lower lip [1]. Since the establishment of the genus *Discogobio* in 1931, research on this group of fish has mainly focused on species descriptions and morphological studies [6,7,8]. Not until the 21st century did research on this group of fish gradually begin to involve phylogenetics [2,9,10]. In recent years, a small number of studies have addressed the physiology and reproductive ecology of this group [11,12,13]. In the field of phylogenetic research, Zhou and Zheng [10] primarily conducted an analysis of the morphological phylogeny of this genus using external morphology and musculoskeletal characteristics. The results indicated that the phylogenetic relationships within the genus reflect the morphological similarities among species. The research on the subfamily Labeoninae in China, based on 16S rRNA, indicated that the genus *Discogobio* is distantly related to the genus *Garra*, which also possesses a sucker disc on the lower lip, with no recent common ancestor between these two genera [9]. It is suggested that the sucker disc is an adaptation to flowing water environments and may not have phylogenetic significance. Zheng et al. [2,4,14] conducted phylogenetic studies on the Labeoninae in China, involving four, four, and eight species of the genus *Discogobio*, respectively. These results showed that the phylogenetic relationships among the various species of *Discogobio* did not reflect morphological similarities. Yang et al. [3] conducted a clade division of the Labeoninae, using five species of *Discogobio* and obtaining results similar to those of Zheng et al. [2]. The focus of the aforementioned molecular studies was on a higher taxonomic level, the subfamily Labeoninae, and only a subset of *Discogobio* species was used as representatives, leading to inconsistencies and incompleteness in the samples used across different studies. Within the literature, there has only been a brief mention of the genus *Discogobio*, and dedicated studies on this genus remain relatively scarce.

*Discogobio* is distributed on the Yunnan–Guizhou Plateau and its adjacent regions, located to the southeast of the Qinghai–Tibet Plateau. Notably, it is exclusively found in the Red River, Yangtze River, and Pearl River. This special distribution pattern may have been shaped by geological events. Within the genus *Discogobio*, except for *Discogobio brachyphysallidos* and *Discogobio yunnanensis*, which are both distributed across different rivers, all other species are only found in the nearby waters where the type species was discovered [1]. *Discogobio yunnanensis* and *D. brachyphysallidos* have similar morphological characteristics and can be distinguished by the size of the exposed scale area on the chest and abdomen [1,15]. These two species exhibit similar morphologies and distribution patterns, which reflect the overall distribution of the genus *Discogobio*. Therefore, studying the phylogeography of these two species can enhance our understanding of the evolutionary processes of the genus *Discogobio*. Recently, five species of *Discogobio* from central Yunnan basin have been studied using RAD sequencing and the results indicated that the presence of the species of *Discogobio* is related to the formation of lakes in central Yunnan [16]. While *D. yunnanensis* and *D. brachyphysallidos* were included in the study by Che et al. [16], samples of these two species were collected from a single river drainage, and their divergences were not fully demonstrated. Thus, it is evident that, despite its unique distribution, no studies have been conducted to address the evolution of this group of species from a genomic perspective, and the genome data of this genus remain unknown. This lack of information has hindered researchers from fully understanding its evolutionary history. Up to now, only the genomes of *Labeo rohita* and *Labeo catla* have been assembled within the subfamily Labeoninae [17,18]. Given that the phylogenetic relationships between *Labeo* and *Discogobio* are relatively distant within the subfamily Labeoninae [3], studies on the population genomics of *Discogobio* still lack a suitable reference genome.

Therefore, we assembled a high-quality chromosome-level genome of *D. brachyphysallidos* in this study using Illumina Hiseq, Oxford Nanopore, and high-throughput chromosome conformation capture (Hi-C) sequencing technologies. We conducted the genome comparisons between *D. brachyphysallidos* and the related species within Cypriniformes. We also performed the whole-genome resequencing of five populations of *D. brachyphysallidos* and *D. yunnanensis* to investigate population structure and demographic history of this group of species. Our data will provide a valuable resource for further studies on genetic background of the genus and species evolution related to the uplift of the Qinghai–Tibet Plateau.

## 2. Results

### 2.1. Genome Assembly and Annotation

**Figure 1:**
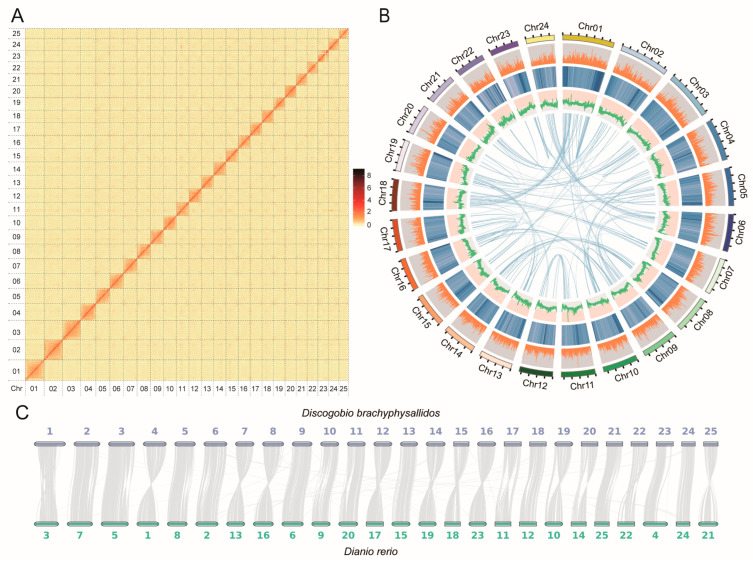
Comparative genomic analyses of *Discogobio brachyphysallidos*. (**A**) Hi-C interaction heatmap for *D. brachyphysallidos* genome showing interactions among twenty five chromosomes (Chr01–25). (**B**) Genomic features of *D. brachyphysallidos*. Tracks from outside to inside are chromosome length (10 Mb), chromosome number, gene count, repeat percentage, GC content, and syntenic blocks on chromosomes. (**C**) Genome collinearity relationship between *D. brachyphysallidos* and *Danio rerio*.

The genome size of *D. brachyphysallidos* was estimated to be 1.18 Gb with a heterozygosity of 0.79% based on k-mer statistics (Supplementary Figure S1). In total, we obtained ~127.61 Gb of clean data of Illumina sequencing with the coverage of 107.91× and ~100.60 Gb of clean data of Nanopore sequencing with the coverage of 83.33× (read number: 4,502,633). For transcriptome data, we obtained ~8.11 Gb of clean data. After data polishing, we obtained ~1.21 Gb genome assembly with contig N50 of 8.63 Mb. We also obtained ~109.17 Gb of clean data of Hi-C (read number: 365,081,953). After incorporating Hi-C linking information, a total of ~1.18 Gb of data were successfully anchored to 25 pseudo-chromosomes, with a scaffold N50 of 42.93 Mb (Table 1, Figure 1A,B). Furthermore, the analysis showed that 98.15% of the assembled bases were located on the chromosomes (Supplementary Table S2). The BUSCO assessment of assembly completeness was up to 94.78% (Table 1). Moreover, the ratio of Illumina sequencing reads was up to 98.64%, and the CEGMA evaluation showed that 97.82% (448/458) of core eukaryotic genes (CEGs) were obtained. The results of collinearity analysis suggested that strong syntenic blocks were found between *D. brachyphysallidos* and the zebra fish, *D. rerio*, and each chromosome showed one-to-one synteny between them (Figure 1C).

Integrating the prediction results obtained by the three methods described above resulted in 30,597 protein-coding genes. These genes contained an average of 8.86 exons, with an average exon length of 2092.72 bp (Supplementary Table S3). Most of the genes originated from transcriptome and orthologous prediction (Supplementary Figure S2), indicating the high prediction quality. The completeness of genome prediction was also assessed by BUSCO, and the results showed that complete BUSCOs was up to 94.15%, including 3365 (92.45%) complete and single-copy BUSCOs (Supplementary Table S4). Repeat sequence analysis indicated that *D. brachyphysallidos* possessed 555.60 Mb of transposable element (TE) sequences and 316.34 kb of tandem repeats, accounting for 46.02% and 0.03% of genome, respectively. Specifically, there were 298.49 Mb of DNA transposons (24.72%) and 256.71 Mb of retroelements (21.26%) (Supplementary Table S5). We also predicted 716 miRNA, 6013 tRNA, 1575 rRNA, 350 snRNA, and 295 pseudogenes for *D. brachyphysallidos*. The information of non-coding RNA sequences is specifically listed in Supplementary Table S6. In total, 93.92% of the protein-coding genes are functionally annotated (Supplementary Table S7).

### 2.2. Gene Family, Phylogenetic Tree, and Divergence Times

**Figure 2:**
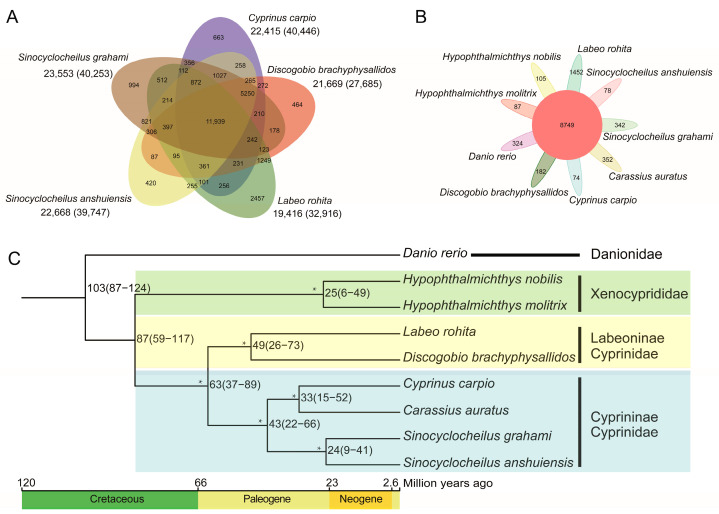
(**A**) Venn diagram of gene family clustering of *D. brachyphysallidos* and another four related species. (**B**) Common and species-specific gene families in *D. brachyphysallidos* and another eight related species. (**C**) Maximum-likelihood tree and divergence time of nine fish species. An asterisk indicates the bootstrap support value of 100 inferred by IQ-TREE.

We identified a total of 32,829 orthogroups in *D. brachyphysallidos* and the other eight species of Cypriniformes, and 285 single-copy orthogroups were also identified in these nine species. In addition, we identified a total of 21,669 orthogroups, and 27,685 genes in these orthogroups for *D. brachyphysallidos* (Figure 2A). Out of these, 182 orthogroups were specific to *D. brachyphysallidos* (Figure 2B). Functional enrichment analyses of specific orthogroups in *D. brachyphysallidos* were conducted, and these orthogroups were mainly enriched in amino acid metabolism and cellular function. A total of 143 and 40 gene families were significantly expanded and contracted in *D. brachyphysallidos*, respectively (Supplementary Figure S3). The functional enrichment of expanded gene families was analyzed using KEGG and GO. The expanded gene families were primarily enriched in the signaling pathways involved in the identification and response to bacterial, viral, and pathogen infections in KEGG. The categories of signaling pathways included NOD-like receptor signaling pathways, notch signaling pathways, and C-type lectin receptor signaling pathway (Supplementary Figure S4). The expanded gene families were mainly annotated in the regulation and negative regulation of viral processes and viral life cycles in biological processes. These gene families were also annotated in aspartic-type endopeptidase, ubiquitin-protein transferase, and olfactory receptor activity in molecular function of GO (Supplementary Figure S5).

To investigate the phylogenetic position of *D. brachyphysallidos*, we constructed a maximum-likelihood phylogenetic tree with a trimmed and concatenated protein sequence alignment from single-copy orthogroups in nine species. The phylogenetic results indicated that *D. brachyphysallidos* was most closely related to *Labeo rohita*, which both belonged to the subfamily Labeoninae. Species of subfamily Cyprininae, namely *Cyprinus carpio*, *Carassius auratus*, *Sinocyclocheilus grahami*, and *Sinocyclocheilus anshuiensis*, formed a lineage, and further formed the sister clade to the subfamily Labeoninae. *Discogobio brachyphysallidos* and *L. rohita* were estimated to have diverged approximately 49 million years ago (Mya) (95% confidence interval, 26–73 Mya). *Sinocyclocheilus grahami* and *S. anshuiensis* diverged approximately 24 Mya (95% confidence interval, 9–41 Mya), *C. carpio* and *C. auratus* approximately 33 Mya (95% confidence interval, 15–52 Mya), and *Hypophthalmichthys molitrix* and *Hypophthalmichthys nobilis* approximately 25 Mya (95% confidence interval, 6–49 Mya) (Figure 2C).

### 2.3. Population Genetic Structure and Demographic History

**Figure 3:**
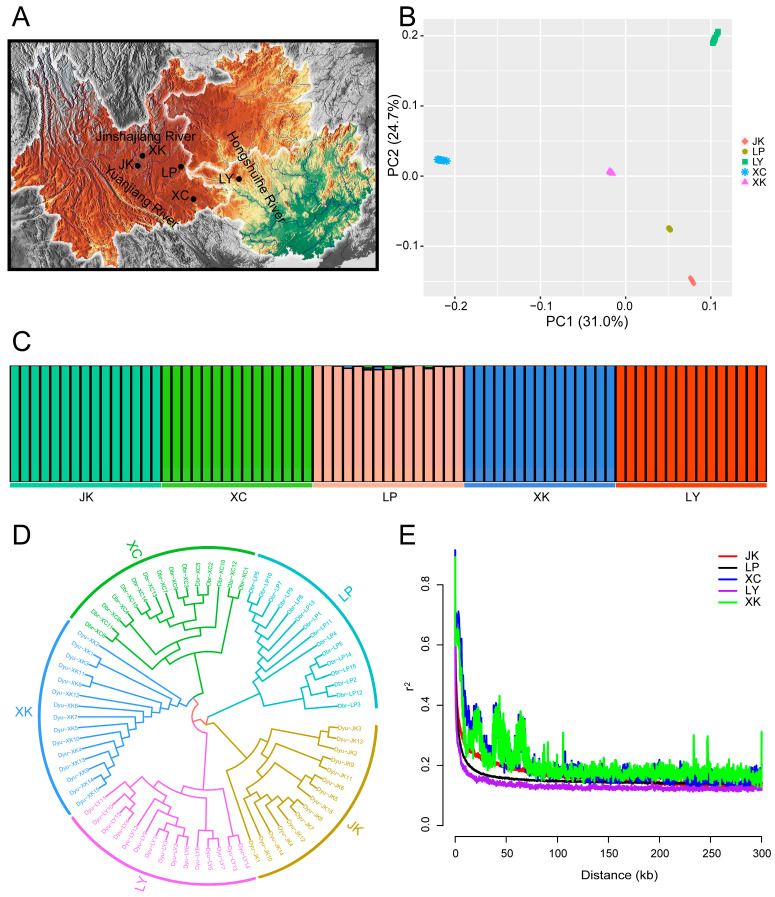
Population genetics analysis for *Discogobio brachyphysallidos* (LP and XC) and *Discogobio yunnanensis* (JK, LY, and XK). (**A**) Geographic distribution of the sampling locations. (**B**) Principal component analysis (PCA) plots of the first 2 components. (**C**) Population structure plots of *D. brachyphysallidos* and *D. yunnanensis*. (**D**) Neighbor-joining phylogenetic tree of individuals based on whole-genome SNP loci. (**E**) Linkage disequilibrium (LD) patterns for the populations. X-axis: physical distances between two SNPs marked in kb; Y-axis: r^2^ used to measure linkage disequilibrium.

We re-sequenced 75 samples of *D. brachyphysallidos* and *D. yunnanensis* from five localities to examine population genetics (Figure 3A). A total of 1.82 Tb of clean data were obtained after quality filtering for all samples. The mapping rates for each sample were 94.63–98.38% in the present study. After mapping, the average coverage depth per sample was estimated as 11.72–28.47 (Supplementary Table S8). A total of 891,303,336 high-quality single nucleotide polymorphisms (SNPs) were identified for all populations. The results of PCA analysis showed that five populations were clustered into five groups (Figure 3B). In the neighbor-joining (NJ) tree, all populations were also clustered into five distinct groups, as shown by the structure analysis (Figure 3C,D). The structure analysis revealed that five populations were divided into five clusters, and the cross-validation error reached its lowest point at K = 5 (Supplementary Figure S6). The decay distance of linkage disequilibrium (LD) was found to be notably small across five different populations. Among these populations, LD decay was relatively higher in JK, LP, and LY, while XC exhibited the lowest decay rate (Figure 3E).

**Figure 4:**
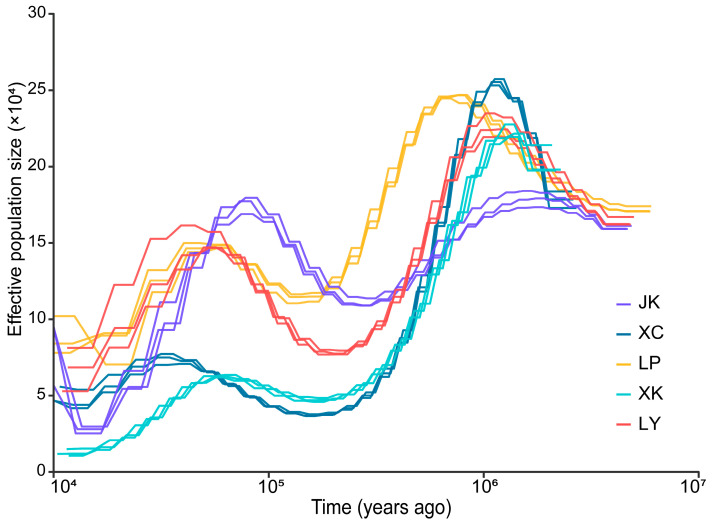
Variation in effective population size and divergence times of five populations of *Discogobio brachyphysallidos* (LP and XC) and *Discogobio yunnanensis* (JK, LY, and XK).

In the analysis of selective sweeps, it was observed that five populations formed five distinct clusters, and hence these five populations were compared pairwise, resulting in a total of ten comparison groups. All the pairwise Fst values between populations ranged from 0.2130 to 0.3485, and the most genetic differentiation was observed between population XC and XK (Supplementary Table S9). Our results indicated that the LP population had the highest pi values, followed by the JK and LY populations. The XC and XK populations displayed relatively lower pi values (Supplementary Table S10). The Tajima’s D estimate produced positive results for all five populations (Supplementary Table S10). The results of ancestral effective population size (*Ne*) showed *Discogobio* experienced two declines in population numbers during the periods of 1.1–0.5 Mya and 0.06–0.02 Mya (Figure 4).

## 3. Discussion

The k_mer analysis indicated that the genome of *D. brachyphysallidos* was a large and complex one and was highly heterozygous. Although the genomes of *L. rohita* and *L. catla* within Labeoninae have been assembled, they have not been assigned to the chromosomes [17,18]. Overall, the genome size of *D. brachyphysallidos* was similar as that of *L. rohita* and *L. catla.* We also compared the genome size of *D. brachyphysallidos* and the diploid relatives within Cypriniformes, and it was found that the genome size of *D. brachyphysallidos* was relatively larger than that of species such as *Acrossocheilus fasciatus* (879.52 Mb), *H. molitrix* (782 Mb), and *H. nobilis* (844 Mb) [19,20]. The same trend was observed for the numbers of protein-coding genes. We predicted 30,597 protein-coding genes in *D. brachyphysallidos*, and a total of 24,900, 24,571, and 24,229 protein-coding genes were predicted for *A. fasciatus*, *H. molitrix*, and *H. nobilis*, respectively. In addition, *D. brachyphysallidos* had the approximately same repeat sequence proportion as the other three species (44.45% in *A. fasciatus*; 41.08% in *H. molitrix*; and 48.24% in *H. nobilis*) [19,20]. It can be seen that *D. brachyphysallidos* had a relatively similar proportion of repetitive sequences, but it had a larger genome size and more protein-coding genes compared with its diploid relatives within Cypriniformes. The increase in phenotypic complexity during evolution may be attributed to more complex genomes [21]. The genus *Discogobio* is characterized by a specialized oral sucker disc on the lower lip, which enables adaptation to life in fast currents. This morphological adaptation may help explain the higher gene count observed in this group of species. Labeoninae was closely related to the species of Cyprininae in the phylogenetic results based on the single-copy genes, which was consistent with the results of [22]. The dating results were in agreement with the previous estimations [19,23]. The diverge time of Labeoninae occurred during the same time period as the strong uplift of the Qinghai–Tibet Plateau. During this period, the elevation of the plateau rose to 2000 m [24]. It is proven that the geological movements of the Qinghai–Tibet Plateau had a significant influence on the origin and evolution of the subfamily Labeoninae, and it was during the strong uplift period that the species of Labeoninae began to diverge.

The result of population structure suggested that five populations of *D. brachyphysallidos* and *D. yunnanensis* from three river drainages were clustered into five groups. Pairwise Fst values, which measure genetic distance between populations, can indicate the level of population differentiation [25]. Based on our analysis, we found that the pairwise Fst values between JK and XC, as well as between JK and LY, fell between 0.20 and 0.25, indicating a moderate level of genetic differentiation. All other pairwise Fst values were observed to be greater than 0.25, indicating a significant level of differentiation [25]. The greatest genetic differentiation was observed between the population XC from the Red River drainage and the population XK from the Yangtze River drainage. This suggests that these two populations with significant differentiation have evolved independently and have become genetically distinct from each other. In the samples collected from these five locations, there are samples of *D. yunnanensis* from its type locality in the Dianchi drainage (XK) and *D. brachyphysallidos* from its type locality in Luoping (LP). According to the current taxonomic framework, *D. yunnanensis* and *D. brachyphysallidos* are distinguished by the size of the exposed scale area on the chest and abdomen. However, the samples identified as these two species do not form two distinct monophyletic groups, indicating the existence of cryptic species within the genus. This suggests that the current taxonomic classification needs to be revised. The positive values of Tajima’s D for all populations suggests that all the populations of *Discogobio* had not experienced recent population expansion [26]. The patterns of the LD decay could be influenced by various factors like natural selection, genetic drift, population structure, and bottlenecks [27]. They can also reveal valuable insights into genetic diversity [28]. In our study, we found that the LD decay was high in all five populations, with the higher levels observed in populations JK, LP, and LY. This indicates that the three populations from the Pearl River drainage have a relatively higher genetic diversity compared with the XC population from the Red River drainage and the XK population from the Yangtze River drainage. This conclusion is supported by the pi values of *Discogobio* populations.

The analysis of the ancestral effective population size revealed that the populations of *Discogobio* have experienced two declines in the history. The times of the two population decreases coincided with the early Pleistocene and Quaternary glaciation. During the middle Tertiary era, the surface of the Qinghai–Tibet Plateau was flattened due to the great climate and environmental changes [24]. Since 3.4 Mya, the Qinghai–Tibet Plateau has experienced significant uplift, leading to the dissection and fragmentation of the primary peneplain [24]. During 1.1–0.6 Mya, the Kunlun-Huanghe movement occurred, elevating the plateau to approximately 3000 m and causing the plateau to enter the glaciation zone on a large scale [29]. At 0.03 Mya, a huge ancient lake (known as the pan-lake period) was formed on the plateau, and the movement caused the huge lake to suddenly flow to the periphery of the plateau [30,31]. The pan-lake dumping incident led to significant environmental changes including deep riverbeds forming around the plateau, and flooding in the middle and lower reaches of the rivers [31]. *Discogobio* possesses an oral sucker on the lower lip, which allows it to adapt to living in fast-flowing environments [1]. The populations entered their respective habitats with the flow of water and evolved separately following the incision and formation of the river. Our results supported that the evolution of this group of species is closely related to the uplift of the Tibet Plateau and the incision of surrounding rivers. In addition, the Quaternary glaciation since the upper Pleistocene (~1.17 Mya) has also led to significant climate change [32]. The first decline in *Discogobio* population occurred during the Kunlun-Huanghe movement, as well as during the first two major glaciations of the Quaternary era (1.17–0.8 Mya and 0.72–0.5 Mya). The second decline in *Discogobio* population coincided with the Last Glaciation (0.07–0.01 Mya). It is indicated that the population number of *Discogobio* was greatly affected by geological movements of the Qinghai–Tibet Plateau and global climate changes during this period. As the geological environment changed, the climate became colder and glaciers expanded, the habitat of the *Discogobio* would have been greatly affected and reduced. This change ultimately resulted in a decline in the population. Therefore, we believe that the decline in *Discogobio* populations was a result of significant geological events of the Qinghai–Tibet Plateau and the Quaternary glaciation. The historical population dynamics of *Discogobio* are largely consistent with those of *Labeo rohita*, which experienced a significant decline after 1 Mya [33]. This timing corresponds closely with the initial population decline observed in *Discogobio* in this study. Furthermore, the subsequent population decline in *Discogobio* corresponds with the decline observed in the eastern population of *Schizothorax oconnori*, which also experienced decline during the Quaternary glaciation. Our findings support population genetics studies of *Schizothorax oconnori*, which indicated that the uplift of the Qinghai–Tibet Plateau and Quaternary climatic oscillations have significantly influenced the population genetics and dynamics of this species [34]. Additionally, the evolution of the genera *Schizothorax* and *Sinocyclocheilus* is considered to be correlated with the uplift of the Qinghai–Tibet Plateau, with both genera exhibiting similar distribution patterns [35,36]. Similar distribution patterns suggest that the evolution and divergence of these species are influenced by the same geological events. Studying the evolution of various species with similar distribution patterns can enhance our understanding of region’s geological history.

## 4. Materials and Methods

### 4.1. Sampling and Sequencing

One sample for whole-genome sequencing was collected from the Nanpanjiang River (upper Pearl River), Luoping, Yunnan Province, China. Muscle tissue was collected for DNA extraction. In order to obtain the chromosome-level genome assembly, three methods were adopted: Illumina paired-end (PE) sequencing, Nanopore sequencing, and Hi-C technology. For Illumina and Hi-C sequencing, the short paired-end (PE) library (350 bp insert size) was constructed and sequenced on the Illumina NovaSeq 6000 platform (Illumina, San Diego, CA, USA). For Nanopore sequencing, the large segment library was premixed with loading beads and then pipetted into a previously used and washed R9 flow cell. The library was sequenced on the ONT PromethION platform with Corresponding R9 cell and ONT sequencing reagents kit (Oxford Nanopore Technologies, Oxford, UK) according to the manufacturer’s instructions. In addition, the transcriptome sequencing was adopted for assisting genome assembly. Samples from the brain, heart, liver, kidney, muscle, and skin were collected for mixed library construction in transcriptome sequencing. A strand-specific library with insert size of 250–350 bp was built and then sequenced using 150 bp paired-end reads on the Illumina Novaseq 6000 sequencing platform.

In order to examine the evolutionary history of the species, we collected 75 individuals of *Discogobio brachyphysallidos* and *Discogobio yunnanensis* from five localities (JK, XC, LP, XK, and LY) for resequencing (Supplementary Table S1). Total DNA was extracted from the fin clips with the Tsingke Genomic DNA Kit (Beijing, China), and sequencing libraries were generated using the NEB Next^®^ Ultra DNA Library Prep Kit for Illumina^®^ (NEB, Ipswich, MA, USA) following the manufacturer’s recommendations. The library preparations were sequenced on the Illumina Novaseq 6000 platform and 150 bp paired-end reads were generated.

### 4.2. Genome Assembly

Firstly, the K-mer analysis was used to estimate the genome size and heterozygosity using the kmer_freq program in the GCE v1.0.2 [37]. De novo genome assembly was performed by the combination of three strategies: initially with software CANU v1.8 [38] correction on clean data, and then with WTDBG2 [39] to assemble the genome sequence, followed by error correction using software Racon v1.4.3 [40] and adjustment by Pilon v1.23 [41]. To evaluate the completeness of the genome assembly, we assessed the alignment ratio to the Illumina sequencing reads. We also conducted CEGMA evaluation and evaluated the integrity of Benchmarking Universal Single-Copy Orthologs (BUSCO). The alignment ratio of Illumina sequencing reads was determined using BWA v0.7.10 [42]. We compared the results with conserved core genes from eukaryotic and actinopterygii databases using CEGMA v2.5 [43] and BUSCO v4.0 [44].

Before chromosome assembly, we first performed a preassembly for error correction of contigs which required the splitting of contigs into segments of 50 kb on average. The Hi-C data were mapped to these segments using BWA, and then the uniquely mapped data were retained to perform assembly by using LACHESIS (http://shendurelab.github.io/LACHESIS/ accessed on 10 November 2021). To estimate the reliability of the genome assembly, MCScanX [45] was used to identify syntenic blocks between *D. brachyphysallidos* and *Danio rerio*.

### 4.3. Repeat Sequences, Non-Coding Gene Prediction

Tandem repeat sequences were predicted using MISA v2.1 [46] and Tandem RepeatFinder v4.09.1 [47]. Transposon elements (TE) were identified by a combination of de novo and homology-based methods. We identified de novo repetitive sequences using RepeatModeler v2.0.1 [48], which automatically executed two repeat finding programs, including RECON v1.08 [49] and RepeatScout [50]. Then, the long terminal repeat sequences (LTR) were identified using both LTRharvest v1.5.9 [51] and LTR finder v1.1 [52]. The high-quality intact LTR and non-redundant LTR libraries were then produced by LTR retriever v2.8 [53]. A non-redundant species-specific TE library was constructed by combining Repbase v19.06 [54], REXdb v3.0 [55], and Dfam v3.2 [56] databases. Final TE sequences were identified and classified by homology search by the library using RepeatMasker v4.10 [57].

The tRNAscan-SE v1.3.1 [58] was used to predict tRNA with eukaryote parameters. Identification of the rRNA genes was conducted by Barrnap v0.9 (https://github.com/tseemann/barrnap/ accessed on 15 November 2021). The miRNA was identified by searching miRBase v21 databases [59]. The snoRNA and snRNA genes were predicted using Infernal v1.1 [60] against the Rfam v12.0 [61] database.

For pseudogene prediction, genBlastA v1.0.4 [62] was first used to scan the whole genomes after masking predicted functional genes. Putative candidates were then analyzed by searching for non-mature mutations and frame-shift mutations using GeneWise v2.4.1 [63].

### 4.4. Protein-Coding Gene Prediction and Functional Annotation

Three methods were used to predict the protein encoding genes—ab initio, homology-based, and transcriptome. Augustus v2.4 [64] and SNAP [65] were used for ab initio prediction, and GeMoMa v1.7 [66] was used for homology-based prediction. Unigenes were assembled by using Trinity v2.11 [67], and then predicted with PASA v2.0.2 [68]. Finally, EVidenceModeler v1.1.1 [69] was used to integrate the prediction results obtained by the above three methods. The completeness of gene prediction was further evaluated using BUSCO. The protein-coding gene annotation was performed with BLAST v2.2.31 [70] by searching the gene ontology (GO), Kyoto Encyclopedia of Genes and Genomes (KEGG), eukaryotic orthologous groups (KOG), Pfam, SWISS-PROT, TrEMBL, EggNOG, and GenBank Non-Redundant (NR) databases.

### 4.5. Gene Family Clustering, Phylogenetic Tree, and Divergence Times Analysis

Protein-coding sequences of the eight species of *Cypriniformes*, namely *C. carpio*, *C. auratus*, *D. rerio*, *H. molitrix*, *H. nobilis*, *L. rohita*, *S. anshuiensis*, and *S. grahami*, were downloaded from NCBI (https://www.ncbi.nlm.nih.gov/ accessed on 13 February 2022) and the China National GeneBank (CNGB, https://db.cngb.org/cnsa/ accessed on 13 February 2022). OrthoFinder v2.5.1 [71] was used to identify the orthologs between these eight species and *D. brachyphysallidos*, using BLAST as the search tool. Single-copy orthologs across all species were selected for gene family and phylogenetic analyses. Proteins of these genes were aligned with MAFFT v7.205 [72] and concatenated to a super alignment matrix. And then the maximum-likelihood tree was constructed using IQ-TREE [73] with the model selected by ModelFinder [74] and 1000 bootstraps.

The divergence time between all species was estimated using MCMCtree in PAML [75], with 20,000 generations and a burn-in of 2000 iterations. Three calibration points from the TimeTree database (http://www.timetree.org/ accessed on 20 February 2022) were adopted: divergence time between *S. anshuiensis* and *D. rerio* (88–125 Mya), *S. anshuiensis* and *C. carpio* (17–51 Mya), and *H. molitrix* and *H. nobilis* (3.5–7.3 Mya).

CAFÉ [76] was used to analyze the expansion and contraction of gene clusters in the *D. brachyphysallidos* genome. The enrichment of gene families in KEGG pathways and GO enrichment analysis were analyzed using clusterProfiler v3.6.0 [77].

### 4.6. Whole-Genome Resequencing and SNP Calling

A total of 75 individuals representing five populations were resequenced by standard procedures on the Illumina HiSeq X Ten platform to yield 150 bp paired-end reads with an insert size around 300 bp. The paired-end reads from each individual were mapped to the genome assembly of *D. brachyphysallidos* using the BWA. Based on the mapping result, duplicated reads were removed using SAMtools v1.9 [78]. SNP and InDel variants were identified using the HaplotypeCaller module in GATK v3.3 [79], generating gVCF for each sample. And then all the gVCF files were merged into the population join genotype. Finally, we obtained the final variant site set after filtered with the following setting: “QD < 2.0||FS > 60.0||MQ < 40.0”—clusterWindowSize 5.

### 4.7. Population Structure and Selective Sweep Analyses

Principal component analysis (PCA) was performed with Eigensoft v6.1.4 [80]. A neighbor-joining tree was constructed using MEGA vX [81] with the p-distance method and with 1000 bootstraps. Genetic structure based on SNP variation was analyzed using ADMIXTURE v1.3.0 [82] with 1–5 ancestral clusters (K). The population differentiation index (Fst), nucleotide polymorphism (π), and Tajima’s D were calculated using VCFtools v0.1.16 [83] with a window size of 100 kb and a step size of 10 kb.

### 4.8. LD Analysis and Demographic Estimation

We calculated the pairwise correlation coefficients (r^2^) between genotypes using PLINK v2 [84]. Recent demographic history was measured by the trend in effective population size (*Ne*) change over time using Pairwise Sequentially Markovian Coalescent (PSMC) v0.6.5 [85]. The estimated generation time (g) was set as 2 years, and the mutation rate was 4 × 10^−9^ mutations per generation, per site [86].

## 5. Conclusions

We first de novo sequenced and assembled a high-quality chromosome-level genome of *D. brachyphysallidos* using Illumina, Nanopore, Hi-C, and RNA sequencing technologies. This assembled genome was 1.21 Gb in length with the contig N50 length of 8.63 Mb. A total of 30,597 protein-coding genes and 8599 non-coding genes were predicted, and 93.92% of the protein-coding genes were annotated. The studies of population genomics revealed that the evolutionary process of the genus *Discogobio* was significantly influenced by the uplift of the Qinghai–Tibet Plateau and the Quaternary glaciation. Our research presents the first high-quality reference genome at the chromosome level within the subfamily Labeoninae, also establishing a foundational dataset for the conservation and molecular breeding of species within this genus. Additionally, our data offer high-resolution genetic information that will facilitate further studies on the genetic background of the genus and the evolutionary dynamics of species in relation to the uplift of the Qinghai–Tibet Plateau.

## Figures and Tables

**Table 1:** Genome assembly statistics of *Discogobio brachyphysallidos*.

Genome Features	Count
Nanopore + Illumina Assembly	
Contig number > 1 kb	1484
Contig N50 (bp)	8,628,411
Contig N90 (bp)	726,229
Max contig size (bp)	37,206,893
Total size (bp)	1,207,250,856
Hi-C assembly	
Scaffold number > 1 kb	1246
Scaffold N50 (bp)	42,928,644
Scaffold N90 (bp)	36,235,350
Max scaffold size (bp)	65,898,982
Contig number	1500
Contig N50 (bp)	8,594,698
Contig N90 (bp)	655,232
Total anchored size (bp)	1,184,958,010
BUSCO actinopterygii (%)	
Complete BUSCOs	94.78
Complete single copy	92.8
Complete duplicated	1.98
Fragmented BUSCOs	0.47
Missing BUSCOs	4.75

## Data Availability

This whole genome assembly project has been deposited at GenBank under the accession number JBDKWK000000000. The Hi-C reads and transcriptome reads were deposited at GenBank under the accession numbers SRR30640985 and SRR30640986. The Nanopore, Hi-C, and resequencing reads were deposited at the China National GeneBank (CNGB) DataBase under the accession number CNP0006276.

## Supplementary Materials

The supporting information can be downloaded at: https://www.mdpi.com/article/10.3390/ijms252413462/s1.
