# Supplementary material for: Chromosome-Level Genome Assembly of Discogobio brachyphysallidos (Teleostei, Cyprinidae) and Population Genomics of the D. brachyphysallidos Complex: Impacts of Geological and Climate Changes on Species Evolution in Southwest China
Source: Int J Mol Sci. 2024 Dec 16;25(24):13462. doi: 10.3390/ijms252413462 (PMC11676654; doi:10.3390/ijms252413462)
Supplement: Supplementary file 1 [file ijms-25-13462-s001.zip › ijms-3296210-supplementary.pdf]

Supplementary Information for

Title: **Chromosome-level genome assembly of *Discogobio brachyphysallidos* (Teleostei, Cyprinidae) and population genomics of the *D. brachyphysallidos* complex: impacts of geological and climate changes on species evolution in Southwest China**

Author(s): Lan-Ping Zheng *, Li-Li Wu, Hua-Ying Sun

Affiliation(s):

College of Chinese Materia Medica, Yunnan University of Chinese Medicine, Yuhua Road 1076, Kunming, China

* Correspondence: zhenglanping@ynucm.edu.cn.

Table S1: Samples information used for the resequencing analysis.

Sampling locality	Abbreviation	Sample number	Drainage
Jinning, Yunnan	JK	15	Nanpanjiang, upper Pearl River
Xichou, Yunnan	XC	15	Lujiang, upper Red River
Luoping, Yunnan	LP	15	Nanpanjiang, upper Pearl River
Kedu, Yunnan	XK	15	Jinshajiang, upper Yangtze River
Lingyun, Guangxi	LY	15	Hongshuihe, upper Pearl River

Table S2: Statistic of chromosomes of *Discogobio brachyphysallidos* using Hi-C technology.

Chromosome id.	Length (bp)
Chr01	81,533,787
Chr02	65,982,349
Chr03	62,867,012
Chr04	56,034,422
Chr05	57,034,493
Chr06	47,876,762
Chr07	47,090,564
Chr08	47,113,273
Chr09	45,614,357
Chr10	46,984,720
Chr11	44,465,514
Chr12	43,941,285
Chr13	43,196,744
Chr14	43,822,623
Chr15	43,179,851
Chr16	42,712,613
Chr17	41,001,496
Chr18	40,381,541
Chr19	43,636,113
Chr20	39,471,457
Chr21	42,701,148
Chr22	44,875,173
Chr23	38,900,729
Chr24	38,270,126
Chr25	36,269,858
Total (Ratio %)	1,184,958,010 (98.15)

Table S3: General statistics of predicted protein-coding genes for *Discogobio brachyphysallidos* and related species.

Species	Number	Average gene length (bp)	Average CDS length (bp)	Average exon length per gene (bp)	Average exon Num per gene	Average intron length per gene (bp)
L. rohita	37,426	15,609.75	1,494.79	1,494.79	8.18	14,114.96

C. carpio	47,917	13,408.21	1,210.90	1,757.54	7.7	11,650.67
D. rerio	25,402	31,112.21	1,625.16	2,521.90	9.73	28,590.30
D. brachyphysallidos	30,597	22,616.50	1,597.64	2,092.72	8.86	20,523.78
S. grahami	45,446	18,577.62	1,571.27	2,214.02	9.66	16,363.60

Table S4: BUSCO assessment results for completeness of gene prediction for *Discogobio brachyphysallidos.*

Total Lineage BUSCOs	3,640
Complete BUSCOs(C)	3,427 (94.15%)
Complete and single-copy BUSCOs(S)	3,365 (92.45%)
Complete and duplicated BUSCOs(D)	62 (1.70%)
Fragmented BUSCOs(F)	38 (1.04%)
Missing BUSCOs(M)	175 (4.81%)

Table S5: Summary of different types of transposable elements in the genome of *Discogobio brachyphysallidos.*

Type	Number	Length	Rate(%)
ClassI:Retroelement	917,404	256,705,242	21.26
ClassI/DIRS	17,459	14,974,688	1.24
ClassI/LINE	88,969	31,652,060	2.62
ClassI/LTR/Copia	5,699	2,219,151	0.18
ClassI/LTR/ERV	14,944	1,652,613	0.14
ClassI/LTR/Gypsy	110,453	49,489,653	4.1
ClassI/LTR/Ngaro	5,463	1,604,155	0.13
ClassI/LTR/Pao	2,864	945,679	0.08
ClassI/LTR/Unknown	664,241	153,145,542	12.69
ClassI/SINE	7,312	1,021,701	0.08
ClassII:DNA transposon	1,116,164	298,485,133	24.72
ClassII/Academ	89	18,799	0
ClassII/CACTA	201,015	45,301,760	3.75
ClassII/Crypton	3,183	466,555	0.04
ClassII/Dada	5,005	509,898	0.04
ClassII/Ginger	4,151	449,972	0.04
ClassII/Helitron	24,594	11,699,553	0.97
ClassII/IS3EU	31,520	9,612,326	0.8
ClassII/Kolobok	56,060	12395440	1.03
ClassII/Maverick	4,013	833,685	0.07
ClassII/Merlin	14,132	2,244,124	0.19
ClassII/Mutator	7,564	1,148,545	0.1
ClassII/P	7,902	2,883,101	0.24
ClassII/PIF-Harbinger	35,359	7,286,156	0.6
ClassII/PiggyBac	9,658	1,978,762	0.16
ClassII/Sola	4,796	1,995,630	0.17
ClassII/Tc1-Mariner	74,140	43,732,841	3.62

ClassII/Unknown	394,180	95,902,736	7.94
ClassII/Zator	2,884	713,440	0.06
ClassII/Zisupton	11,953	1,754,328	0.15
ClassII/hAT	223,966	57,557,482	4.77
Unknown	1,464	404,489	0.03
Unspecified	2	143	0
Total	2,035,034	555,595,007	46.02

Table S6: Summary statistics of non-coding RNA annotation for *Discogobio brachyphysallidos*

Type		Copy number	Average length(bp)	Total length(bp)
miRNA		716	85.05	60,894
tRNA		6,013	75.97	456,803
rRNA	rRNA	1,575	113.19	178,268
	18S	7	1,164.86	8,154
	28S	3	3,431.67	10,295
	5.8S	3	127.33	382
	5S	1,562	102.07	159,437
snRNA	snRNA	350	137.62	48,167
	Splicing	245	128.74	31,540
	CD-box	64	144.72	9,262
	HACA-box	35	168.51	5,898
	scaRNA	6	244.5	1,467

Table S7: Functional annotation of protein-coding genes for *Discogobio brachyphysallidos*.

Annotation Database	Annotated Number	Annotated Ratio (%)
GO	23,852	77.96
KEGG	24,894	81.36
KOG	16,875	55.15
Pfam	25,300	82.69
Swissprot	24,409	79.78
TrEMBL	28,685	93.75
eggNOG	24,204	79.11
nr	27,402	89.56
All	28,738	93.92

Table S8: Data quality overview for all re-sequenced samples.

Samples	Total Reads	Clean Reads	Clean bases (bp)	Mapped Reads	Mapped rate (%)	>Q30 %	Average depth	Coverage > 4X
Dbr_LP1	217,839,988	215,534,566	32,156,862,831	211,128,494	97.96	90.01%	23.23	91.11%
Dbr_LP2	223,630,328	221,616,646	33,071,977,227	217,819,893	98.29	90.78%	24.19	91.08%
Dbr_LP3	226,099,286	223,746,534	33,412,464,261	219,726,171	98.20	90.28%	23.95	90.74%
Dbr_LP4	145,629,632	144,417,290	21,560,040,096	141,491,526	97.97	90.52%	16.04	89.16%

Dbr_LP5	139,323,314	137,898,640	20,591,258,810	135,470,920	98.24	91.14%	14.74	87.08%
Dbr_LP6	144,852,508	143,493,408	21,428,054,000	140,688,226	98.05	92.34%	15.41	87.20%
Dbr_LP7	164,802,996	163,293,316	24,387,083,283	160,392,217	98.22	90.22%	17.95	88.95%
Dbr_LP8	142,690,132	141,172,144	21,070,366,708	138,690,068	98.24	90.85%	15.33	87.70%
Dbr_LP9	151,471,394	150,069,160	22,411,319,487	147,510,183	98.29	91.83%	16.08	89.09%
Dbr_LP10	148,053,900	146,676,364	21,897,415,800	143,759,059	98.01	91.93%	15.53	88.12%
Dbr_LP11	173,457,846	171,816,446	25,653,037,125	168,651,447	98.16	92.08%	17.96	89.10%
Dbr_LP12	146,749,432	145,329,358	21,694,803,786	142,824,331	98.28	90.93%	15.88	87.35%
Dbr_LP13	135,388,174	134,193,718	20,035,389,033	132,026,069	98.38	90.55%	14.91	86.91%
Dbr_LP14	172,861,872	171,334,508	25,579,739,093	167,933,781	98.02	90.55%	19.16	89.18%
Dbr_LP15	154,388,048	152,895,744	22,822,662,943	150,143,962	98.20	90.73%	16.70	87.84%
Dbr_XC1	240,776,500	236,899,760	35,324,316,726	232,007,503	97.93	87.50%	26.27	83.67%
Dbr_XC2	211,446,534	209,470,452	31,215,211,309	204,989,130	97.86	90.44%	23.22	82.87%
Dbr_XC3	211,996,778	209,881,162	31,301,726,025	205,592,186	97.96	91.54%	23.04	82.55%
Dbr_XC4	145,962,198	144,647,692	21,570,204,994	141,571,206	97.87	91.45%	16.10	79.79%
Dbr_XC5	188,691,750	186,858,928	27,847,402,978	182,542,257	97.69	92.19%	19.92	81.45%
Dbr_XC6	134,462,264	133,250,544	19,883,586,621	130,745,475	98.12	92.12%	14.9	78.19%
Dbr_XC7	187,995,192	185,929,410	27,723,849,273	182,113,953	97.95	89.32%	20.83	81.93%
Dbr_XC8	165,888,976	164,403,214	24,508,802,731	160,959,671	97.91	92.38%	17.84	80.92%
Dbr_XC9	202,349,956	200,413,684	29,892,959,339	196,593,132	98.09	90.58%	22.33	82.41%
Dbr_XC10	156,062,804	154,851,692	23,097,837,872	151,873,748	98.08	90.54%	18.10	81.12%
Dbr_XC11	169,943,924	168,420,946	25,109,485,737	164,963,817	97.95	92.54%	18.45	80.22%
Dbr_XC12	206,555,106	204,457,850	30,481,398,370	200,406,146	98.02	91.23%	22.51	82.33%
Dbr_XC13	158,058,396	156,274,128	23,300,731,847	153,324,042	98.11	89.38%	18.1	80.08%
Dbr_XC14	158,802,838	157,329,622	23,443,765,019	154,201,104	98.01	90.08%	18.29	80.34%
Dbr_XC15	182,228,452	180,388,620	26,875,767,378	176,561,735	97.88	91.36%	19.85	81.44%
Dyu_JK1	193,737,266	192,040,294	28,640,110,443	188,865,632	98.35	90.77%	21.51	88.69%
Dyu_JK2	217,752,678	215,681,490	32,185,547,470	211,955,159	98.27	90.41%	24.28	89.11%
Dyu_JK3	187,503,472	185,114,898	27,608,653,627	181,533,937	98.07	89.52%	20.88	88.36%
Dyu_JK4	120,163,098	118,731,670	17,711,216,732	116,414,099	98.05	90.60%	13.59	84.53%
Dyu_JK5	129,197,436	127,839,378	19,069,460,458	125,261,462	97.98	90.51%	14.85	85.68%
Dyu_JK6	122,577,466	121,070,656	18,061,234,393	118,530,366	97.90	89.19%	13.99	84.87%
Dyu_JK7	106,609,150	105,322,160	15,717,456,599	103,348,288	98.13	89.70%	12.30	82.93%
Dyu_JK8	123,546,096	122,132,892	18,214,908,947	119,802,221	98.09	89.72%	14.20	85.36%
Dyu_JK9	158,883,194	157,100,062	23,437,223,236	154,192,055	98.15	90.64%	17.25	87.17%
Dyu_JK10	129,965,702	128,578,064	19,176,525,645	125,992,437	97.99	89.48%	14.90	85.79%
Dyu_JK11	138,612,894	137,167,168	20,446,442,764	134,433,529	98.01	90.95%	15.78	86.15%
Dyu_JK12	131,507,256	130,166,208	19,415,209,720	127,558,859	98.00	90.02%	14.79	85.97%
Dyu_JK13	167,637,378	165,983,064	24,765,402,154	162,802,928	98.08	92.30%	17.44	86.88%
Dyu_JK14	148,137,350	146,663,670	21,879,942,971	143,813,125	98.06	90.48%	16.70	86.54%
Dyu_JK15	145,978,170	144,204,044	21,499,376,750	141,588,831	98.19	88.38%	16.70	86.04%
Dyu_LY1	256,794,368	254,319,214	37,950,727,576	249,253,602	98.01	90.28%	28.47	86.45%
Dyu_LY2	211,971,178	209,755,700	31,294,972,008	205,412,730	97.93	90.45%	23.47	85.15%
Dyu_LY3	218,498,562	216,698,698	32,342,329,526	212,537,101	98.08	91.43%	24.51	85.89%

Dyu_LY4	134,077,342	132,873,218	19,836,602,682	130,143,801	97.95	91.00%	15.57	80.85%
Dyu_LY5	158,137,930	156,598,146	23,376,969,887	152,912,534	97.65	90.56%	17.41	82.96%
Dyu_LY6	165,390,258	163,846,288	24,445,530,859	160,594,701	98.02	90.78%	18.54	83.12%
Dyu_LY7	173,520,430	171,828,038	25,633,887,447	167,001,398	97.19	90.75%	18.82	84.08%
Dyu_LY8	149,742,568	148,336,382	22,148,889,664	144,135,029	97.17	91.18%	16.13	82.51%
Dyu_LY9	155,921,294	154,553,054	23,066,201,568	151,158,158	97.8	90.22%	17.78	82.99%
Dyu_LY10	175,894,116	174,225,664	26,007,129,747	169,207,827	97.12	89.84%	19.19	83.56%
Dyu_LY11	157,145,630	155,735,300	23,256,159,185	152,794,587	98.11	91.69%	16.78	82.18%
Dyu_LY12	168,131,564	166,626,704	24,852,139,421	157,682,671	94.63	90.77%	17.95	83.31%
Dyu_LY13	146,348,372	144,810,094	21,618,283,018	141,611,962	97.79	90.93%	15.77	82.03%
Dyu_LY14	138,108,910	136,792,018	20,420,730,470	133,292,404	97.44	91.54%	14.96	81.61%
Dyu_LY15	138,989,332	137,315,716	20,497,352,388	134,069,821	97.64	90.74%	15.27	81.21%
Dyu_XK1	222,463,370	219,939,612	32,544,053,893	214,857,249	97.69	88.23%	23.94	82.75%
Dyu_XK2	252,861,296	250,468,442	37,222,582,430	244,775,739	97.73	90.10%	28.00	83.80%
Dyu_XK3	242,346,936	240,241,682	35,722,254,321	235,078,571	97.85	93.06%	24.69	83.03%
Dyu_XK4	138,315,368	136,955,608	20,336,015,784	133,899,294	97.77	89.55%	15.67	78.72%
Dyu_XK5	143,483,142	142,065,996	21,071,238,930	137,708,517	96.93	89.25%	16.01	78.53%
Dyu_XK6	153,013,632	151,532,934	22,506,640,701	147,918,199	97.61	89.82%	17.03	79.22%
Dyu_XK7	150,496,840	149,133,456	22,138,549,727	145,801,623	97.77	90.13%	16.82	79.50%
Dyu_XK8	148,117,242	146,594,928	21,694,381,270	143,365,415	97.8	90.12%	16.18	78.91%
Dyu_XK9	134,636,944	133,244,290	19,697,162,001	129,960,793	97.54	89.24%	14.87	77.50%
Dyu_XK10	136,852,668	135,570,936	20,124,405,656	132,283,490	97.58	89.97%	15.39	78.48%
Dyu_XK11	150,708,402	149,340,378	22,224,369,071	145,757,254	97.60	90.08%	17.00	79.46%
Dyu_XK12	149,742,458	148,363,920	22,009,237,884	145,046,157	97.76	89.55%	16.77	79.49%
Dyu_XK13	137,579,090	136,171,702	20,225,513,530	133,166,194	97.79	89.80%	15.72	77.96%
Dyu_XK14	104,332,766	103,195,264	15,279,009,600	100,756,294	97.64	89.87%	11.72	72.61%
Dyu_XK15	115,230,218	113,700,572	16,835,288,829	110,714,464	97.37	88.40%	12.86	74.81%

Table S9: Pairwise Fst value among populations.

Populations	JK	XC	LP	XK	LY
JK		0.2496	0.2747	0.2660	0.2130
XC			0.3361	0.3485	0.2533
LP				0.3397	0.3062
XK					0.2724
LY					

Table S10: The results of sweep analysis of populations.

Populations	pi	Tajima'D
JK	0.00012596	0.42988572
XC	0.00004840	0.27303696
LP	0.00034465	1.76877751

XK	0.00004300	0.22450519
LY	0.00009280	0.21946162

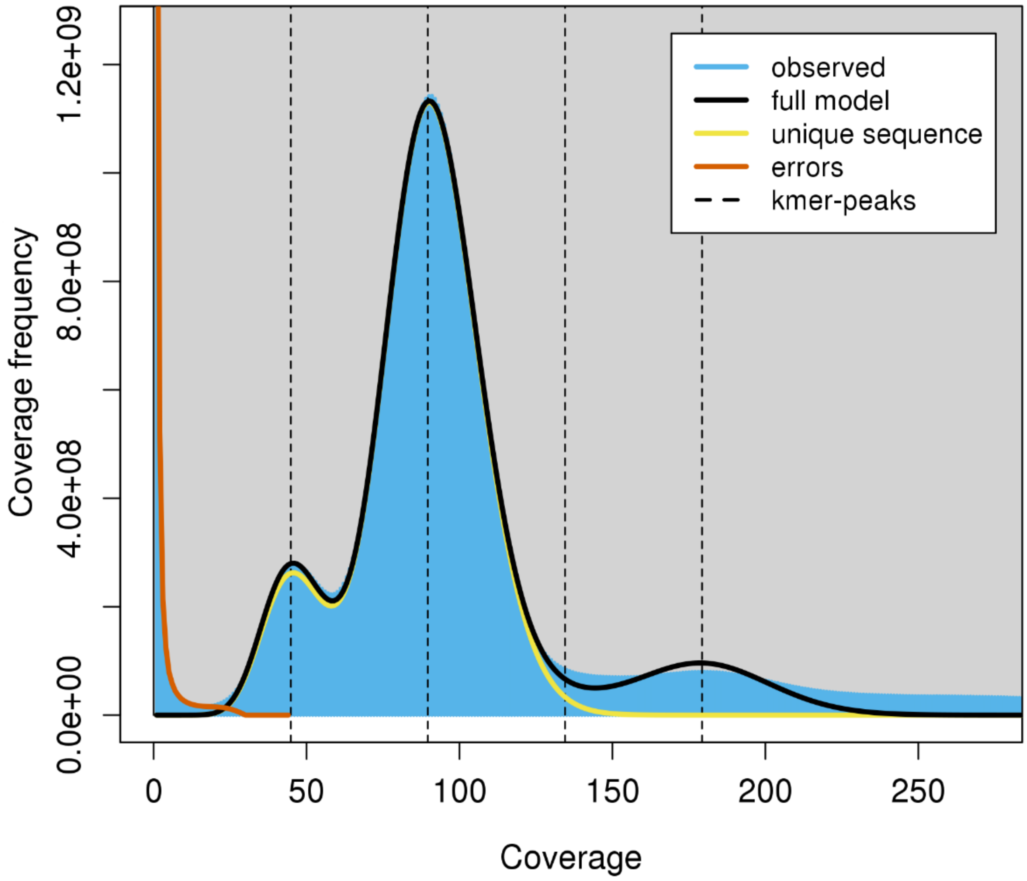

Supplementary Figure S1: Frequency distribution of depth of *K-mer* = 19 in genome survey of *Discogobio brachyphysallidos*.

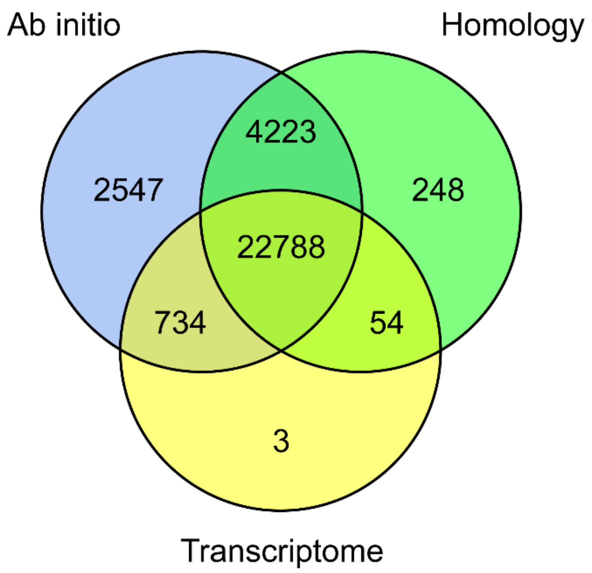

Supplementary Figure S2: The number of integrated genes derived from the three prediction methods

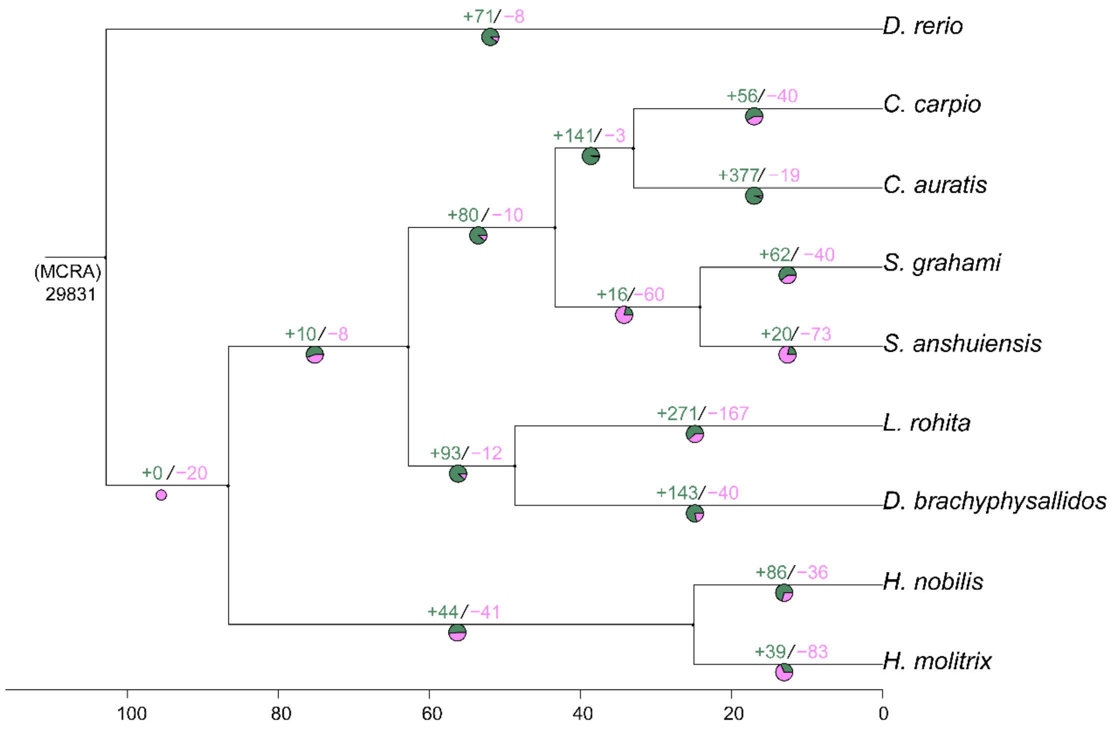

Supplementary Figure S3. The number of expanded and contracted gene families deduced using cafe for each branch.

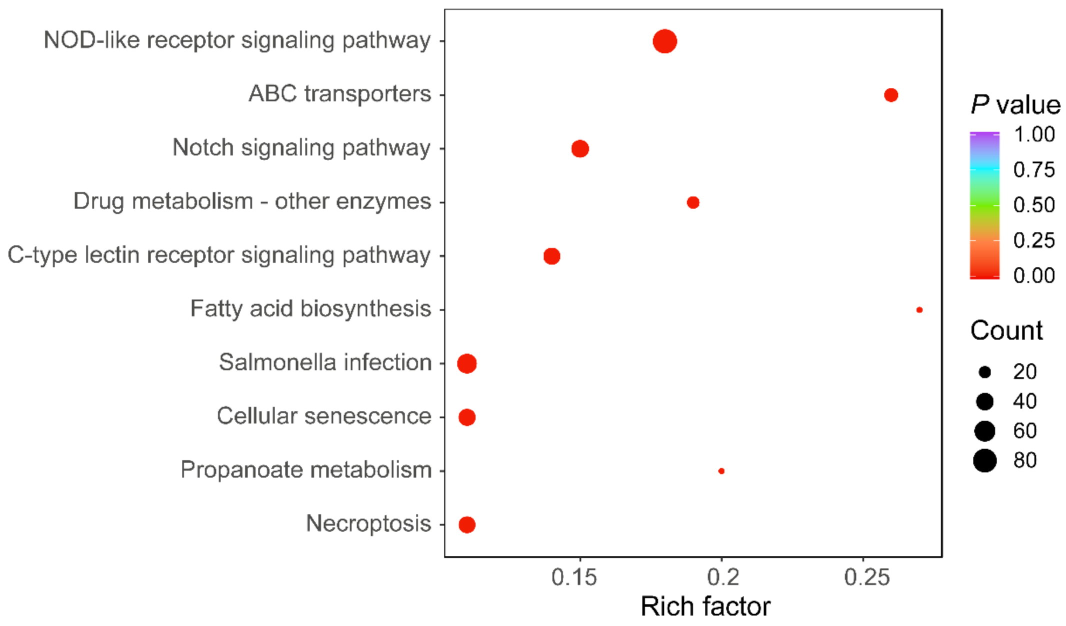

Supplementary Figure S4. KEGG pathway enrichment analyses for expansion gene families for *Discogobio brachyphysallidos*.

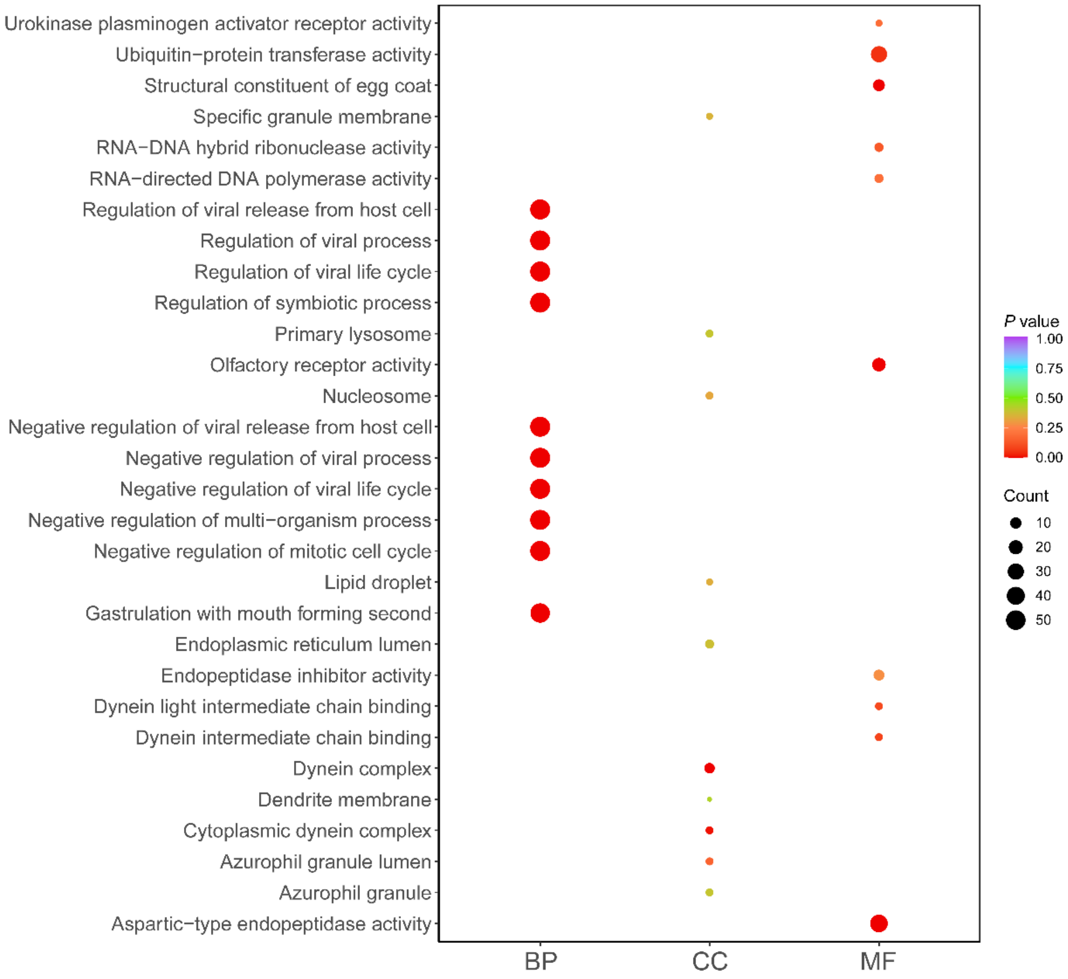

Supplementary Figure S5. GO enrichment analyses for expansion gene families for *Discogobio brachyphysallidos.*

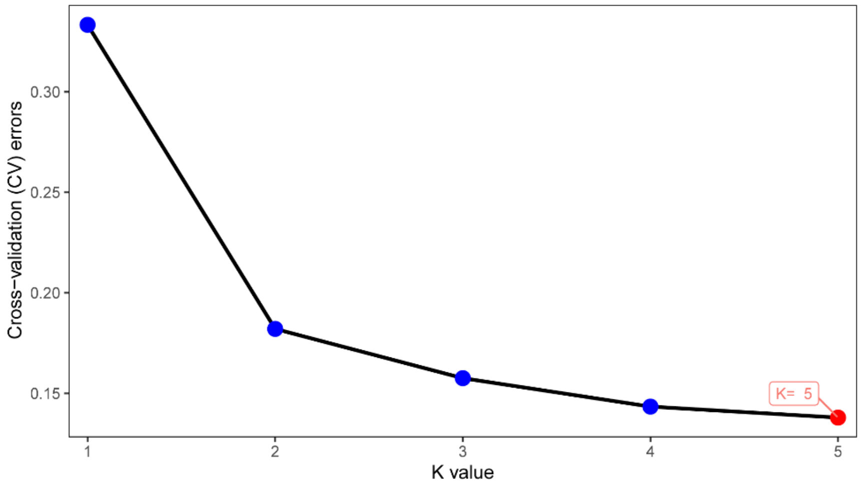

Supplementary Figure S6. Graphs of cross-validation (CV) error against the number of clustered populations (K). The CV error reached the minimum value when K=5.
